# The Role of Toluidine Blue Vital Staining in the Diagnosis of Oral Mucosal Lesions in Dental Practice: A Narrative Review

**DOI:** 10.7759/cureus.112328

**Published:** 2026-07-09

**Authors:** M Saranya, Veeran Veeravarmal, Palanisamy Gowri Shankar, G Harshini, Thangadurai Maheswaran, Vadivel Ilayaraja, Sivanesan Sathiya Narayana Murthy

**Affiliations:** 1 Oral and Maxillofacial Pathology, Vivekanandha Dental College for Women, Unjanai, IND; 2 Oral and Maxillofacial Pathology, Government Dental College and Hospital, Cuddalore, IND; 3 Oral and Maxillofacial Pathology, RVS Dental College and Hospital, Coimbatore, IND; 4 Oral and Maxillofacial Pathology, Sri Ramakrishna Dental College and Hospital, Coimbatore, IND; 5 Oral and Maxillofacial Pathology, Adhiparasakthi Dental College and Hospital, Melmaruvathur, IND; 6 Anatomy, Shri Sathya Sai Medical College and Research Institute, Nellikuppam, IND

**Keywords:** adjunctive diagnostic tools, dysplasia, early diagnosis, oral cancer, oral cancer screening, oral leukoplakia, oral potentially malignant disorders, toluidine blue, vital staining

## Abstract

Oral cancer imposes a disproportionate global health burden, with persistently poor survival rates primarily attributable to late-stage diagnosis. oral potentially malignant disorders (OPMDs) are identifiable and clinically actionable precursors to the development of most oral squamous cell carcinomas (OSCCs). However, accurate risk stratification in routine dental practice remains a challenge. Toluidine blue (TB) vital staining, a cationic, acidophilic, metachromatic dye with selective affinity for nucleic acid-rich dysplastic and malignant epithelium, has been employed as a chairside adjunctive diagnostic tool for more than four decades. This narrative review synthesizes the current evidence on the mechanism of TB staining, its diagnostic accuracy, and its utility within the broader landscape of adjunctive tools available to dental practitioners. TB demonstrates a pooled sensitivity of 71% to 95% and specificity of 68% to 81%, with its diagnostic odds ratio consistently exceeding that of clinical visual examination. Compared with light-based adjunctive tools, TB exhibits superior specificity to chemiluminescence and autofluorescence, although its sensitivity is lower than that of both techniques. The integration of TB into a multimodal diagnostic protocol, combining clinical examination, staining, and, where indicated, histopathological confirmation, optimizes the detection of high-risk lesions, guides biopsy site selection, and supports surgical-margin assessment. Known limitations include false positives from inflammatory conditions, false negatives in hyperkeratotic lesions, and operator-dependent interpretation. Emerging technologies, such as reflection spectroscopy, artificial intelligence-assisted image analysis, and combined multimodal platforms, offer potential solutions to address these constraints. Dental practitioners who incorporate TB into a structured clinical workflow, interpreted alongside the full clinical picture, are well positioned to improve early detection and reduce diagnostic delay, which remains a major determinant of oral cancer mortality.

## Introduction and background

Oral cancer ranks among the most epidemiologically consequential and clinically challenging malignancies encountered in dental practice [1,2]. Over 377,000 new cases are diagnosed globally each year, with a disproportionate burden in South and Southeast Asia, where it is the leading cancer in males [1,3]. Despite the oral cavity being one of the most accessible sites in the human body for routine clinical examination, the disease is persistently detected at late stages, with reported five-year survival rates as low as 40-50% for advanced presentation compared with rates exceeding 80% when identified early [1,4]. This paradox of accessibility and late diagnosis reflects a fundamental deficit in early recognition rather than treatment capacity, reinforcing the clinical urgency of improving frontline detection competency [1,2].

The carcinogenic progression from histologically normal mucosa to oral squamous cell carcinoma (OSCC) is not instantaneous but proceeds through a recognizable spectrum of oral potentially malignant disorders (OPMDs), including leukoplakia, erythroplakia, oral submucous fibrosis, and oral lichen planus, each associated with a quantifiable risk of malignant transformation [5-7]. The window of opportunity for intervention during the OPMD phase, particularly when epithelial dysplasia is present, is clinically actionable but is often missed [1,7]. Visual and tactile examination, although endorsed as the primary screening modality, is limited by considerable inter-examiner subjectivity and is insufficient to distinguish high-risk lesions from low-risk lesions, especially in the early or occult stages [2,8]. Histopathological examination of biopsy specimens remains the diagnostic gold standard but is limited by invasiveness, procedural complexity, patient anxiety, and cost, which reduce its use in primary care settings [1,4].

Adjunctive diagnostic tools, including vital staining, light-based detection systems, oral brush cytology, and spectroscopy, have been developed to bridge this gap [2,8]. Among these, toluidine blue (TB) vital staining is among the most extensively studied and widely employed in dental practice. This narrative review examines the evidence for TB staining in the diagnosis of oral mucosal lesions, covering its mechanistic basis, diagnostic accuracy, comparative performance against other adjunctive tools, integration into a multimodal diagnostic framework, limitations, and emerging technologies that may help define its future role.

## Review

Oral cancer burden and the rationale for vital staining

Oral cancer constitutes a formidable global health burden and occupies an especially alarming position in Asian countries, where it ranks among the most common malignancies and accounts for more than 25% of all new cancer diagnoses annually [1]. Worldwide, over 377,000 new oral cancer cases were recorded in 2020, with a disproportionately high incidence among males and a troubling trend toward younger patients being affected [3]. Oral cancer is the sixth most common cancer worldwide, as part of the head and neck cancer group, and approximately 657,000 new cases are projected globally each year [2]. Despite decades of advancements in oncology, the overall five-year survival rate remains unsatisfactory, at approximately 50% across all stages [4]. When detected at a localized stage, survival can exceed 80%; however, the rate collapses to below 20% when regional lymph nodes are involved, underscoring that diagnostic timing is the most consequential determinant of patient outcome [3].

The predominant histological subtype, OSCC, arises in the mucosal lining of the oral cavity and frequently develops from a recognizable continuum of OPMDs [1]. In 2020, the WHO Collaborating Centre for Oral Cancer convened an expert consensus that formally classified OPMDs as including leukoplakia, erythroplakia, proliferative verrucous leukoplakia, oral lichen planus, oral submucous fibrosis, and additional recently recognized entities such as oral lichenoid lesions and chronic graft-versus-host disease oral manifestations [5]. Oral leukoplakia, the most commonly encountered OPMD, has a pooled malignant transformation proportion of 6.64% (95% CI 5.21-8.21) across 55 studies and 41,231 patients [7]. This risk escalates substantially in the presence of non-homogeneous clinical morphology (relative risk, 4.23), epithelial dysplasia (relative risk, 2.75), large lesion size, tobacco use, and lingual location, with leukoplakias on the lateral border of the tongue showing a malignant transformation rate of 12.71% [7]. A meta-analysis restricted to the preceding five years reported an even higher pooled malignant transformation proportion of 9.8% [6], emphasizing the dynamic and contextually determined nature of malignant risk.

The rationale for adjunctive diagnostic tools emerges from the fundamental gap between the accessibility of the oral cavity and the prevailing late-stage presentation of oral cancers. Visual and tactile examinations, although broadly recommended by the WHO, are limited by subjective interpretation, and clinicians, particularly general dental practitioners, frequently miss early or occult lesions, even within a fully accessible anatomical region [2]. Tissue biopsy followed by histopathological examination remains the gold standard for definitive diagnosis; however, patient reluctance related to fear, discomfort, cost, and procedural anxiety creates substantial diagnostic delays [1]. Therefore, minimally invasive chairside tools that can identify high-risk areas and guide biopsy site selection are a clinical necessity rather than a supplementary convenience [8]. TB vital staining, which has been used for over four decades, represents the best-evaluated and most widely deployed adjunct in dental practice.

Mechanism, clinical indications, and diagnostic accuracy of TB

TB, chemically classified as tolonium chloride or Basic Blue 17, is a phenothiazine cationic, acidophilic, and metachromatic dye [9]. Its selectivity for dysplastic and malignant tissue is based on two principal mechanisms: a high affinity for nucleic acids (DNA and RNA), whose content is amplified in rapidly proliferating neoplastic cells, and the structural property of wider intracellular canals in dysplastic epithelium that facilitate enhanced dye penetration and retention [10]. Spectroscopic investigations have confirmed that TB interacts with genomic DNA at physiological pH (7.4) through a specific monomeric binding pattern, whereas its interaction with proteins under equivalent physiological conditions is negligible, providing a molecular basis for its tumor selectivity [9]. The dye does not cross the intact plasma membranes of normal cells; in malignant epithelium, the amplified nucleic acid content and altered intracellular architecture allow preferential retention, yielding a characteristic royal blue or dark purple staining pattern [8].

The clinical staining protocol is straightforward and feasible in dental settings. Following water rinsing to clear debris, the oral cavity is pretreated with 1% acetic acid for 20 s to remove salivary and bacterial pellicles, rinsed with 1% TB solution for 20-30 s, and then treated with another acetic acid rinse to remove residual dye [11]. Areas of dark blue retention are recorded and photographed using a digital camera. Clinically, TB is most appropriate as a screening adjunct for high-risk patients, for identifying occult lesions not visible under standard incandescent light, and, critically, for guiding biopsy site selection in large or multifocal lesions, where clinicians face field cancerization challenges [11]. A study evaluating TB's impact on biopsy site selection demonstrated that specialist consensus improved significantly in 60% of cases when TB-stained images were used compared with unstained images, suggesting that the dye reduces inter-observer variability in an inherently subjective decision [11].

The diagnostic performance of TB has been quantified in several systematic reviews. A meta-analysis of 29 studies reported a pooled sensitivity of 71.4% (95% CI 60.7-80.2%), specificity of 81.2% (95% CI 67.9-89.7%), and diagnostic OR of 7.017 (95% CI 4.544-10.836), with an area under the summary receiver operating characteristic (SROC) curve of 0.766, reflecting moderate-to-good discriminatory accuracy [12]. Subgroup analysis revealed that TB performs markedly better in detecting severe dysplasia or frank carcinoma than when applied to all dysplastic categories indiscriminately, with higher-grade lesions yielding significantly elevated sensitivity, whereas specificity marginally declined [12]. A diagnostic test accuracy study in South Asian primary care settings reported sensitivity and specificity of 94.6% and 80.0%, respectively, among 162 patients, with the strongest TB positivity correlating with moderate-to-severe epithelial dysplasia and OSCC histopathological categories [3]. A hospital-based Indian study similarly reported a sensitivity of 92.6% and specificity of 67.9%, with an overall diagnostic accuracy of 80% [13]. These results confirm that TB staining consistently surpasses clinical visual examination alone and that its false-positive rate, principally attributable to inflammatory ulcerations and hyperkeratotic surfaces, is a known and manageable limitation rather than a disqualifying deficiency [2].

Comparative evaluation of adjunctive tools in oral cancer diagnosis

TB does not act in isolation. A spectrum of adjunctive tools, including autofluorescence imaging, chemiluminescence (CL), narrowband imaging (NBI), and oral brush cytology, has been evaluated in direct and network comparisons, and their relative performance profiles differ in ways that have direct implications for clinical decision-making.

A systematic review and meta-analysis of 29 studies demonstrated that TB maintains a higher specificity (0.809) and diagnostic OR (10.565) than CL (specificity, 0.345; DOR, 5.203), despite CL achieving a modestly higher sensitivity (0.841 vs. 0.659) [12]. This reciprocal pattern is clinically significant: CL casts a wide net with few false negatives but generates excessive false-positive referrals and biopsies, whereas TB's superior specificity makes it more useful for identifying high-risk areas with greater confidence [14]. A bivariate meta-analysis examining CL confirmed an area under the SROC curve of 0.743, reflecting moderate diagnostic accuracy, and explicitly concluded that CL alone is insufficient as a replacement for conventional clinical examination [14].

Autofluorescence imaging yields higher sensitivity (86-91%) than TB, but at the cost of substantially reduced specificity, with pooled specificity estimates as low as 15-72% across the included studies [15]. The diagnostic OR for autofluorescence was 8.197 (area under SROC = 0.815), showing similar overall discrimination to that of TB, but with a fundamentally different error profile: a high false-positive rate that risks overdiagnosis and patient anxiety [15]. A clinicopathological evaluation comparing all three modalities in 126 patients found that in the detection of dysplastic lesions, autofluorescence showed 84.1% sensitivity but only 15.3% specificity, CL showed 77.3% sensitivity and 27.8% specificity, whereas TB yielded 56.8% sensitivity and 65.8% specificity; crucially, when the three tools were used in combination, specificity improved substantially [16].

A network meta-analysis encompassing six diagnostic modalities found that NBI demonstrated statistical superiority in sensitivity, specificity, negative predictive value, and overall accuracy compared with autofluorescence, CL, cytology, TB, and visual examination; however, the evidence base for NBI remains limited to two or three studies, and its interpretation should be cautious [10]. Acetic acid staining, evaluated as a cheaper alternative to TB, showed a sensitivity of 100% but a specificity of only 13.3%, making it far inferior to TB in distinguishing dysplastic from non-dysplastic lesions [17]. A broader systematic review confirmed that non-invasive tools, including TB, exhibit a consistent pattern of high sensitivity but suboptimal specificity, with TB achieving mean values of 77.26% and 74.37% across six included studies [4].

Table 1 summarizes the comparative diagnostic performances of the principal adjunctive tools evaluated in the included studies [2,3,10,12,14,15,17,18].

**Table 1:** Comparative diagnostic performance of adjunctive tools for detecting oral potentially malignant disorders and oral cancer. AUC: Area under the summary receiver operating characteristic curve; DOR: Diagnostic odds ratio.

Adjunctive tool	Pooled sensitivity	Pooled specificity	DOR/AUC	Key advantage	Key limitation
Toluidine blue	71-95%	68-81%	DOR, 7.0-10.6	High specificity; guides biopsy site selection	False positives in inflammatory lesions
Autofluorescence	83-91%	15-72%	AUC, 0.815	High sensitivity; non-contact technique	Low specificity; requires a dedicated device
Chemiluminescence	83-90%	30-43%	AUC, 0.743	Good sensitivity; portable	Very low specificity; requires a dark room
Narrow-band imaging	~90%	~95%	Highest ranked in network meta-analysis	Superior overall diagnostic accuracy	Very limited evidence base
Oral brush cytology	72-100%	89-100%	High specificity	Non-invasive sampling	Does not replace biopsy
Acetic acid	100%	13%	Poor specificity	Inexpensive	Clinically unreliable

Vital staining in the multimodal diagnostic framework

The clinical utility of TB is best understood not as a standalone diagnostic verdict but as an integrated component of a structured, multimodal diagnostic pathway. The diagnostic process in dental practice ideally proceeds from a thorough visual and tactile examination through risk stratification and the application of adjunctive tools, culminating, when indicated, in the histopathological examination of a targeted biopsy specimen [1]. Within this framework, TB occupies a complementary position that enhances, rather than replaces, the primary examination.

Several properties make TB particularly well-suited for this integrative role. Its ability to stain positively, even in clinically innocuous-looking lesions that harbor significant dysplasia, underpins its value in identifying occult or underestimated lesions [3]. General dental practitioners encounter oral mucosal lesions during routine examinations; however, many lack the specialist experience necessary to confidently stratify lesions according to their risk of malignancy. TB staining provides an objective, chairside indicator that can prompt timely referral for a targeted biopsy [2]. In primary healthcare settings in Sri Lanka, capacity development workshops enabled dental surgeons to apply TB reliably in peripheral clinics, demonstrating that the technique is transferable to low-resource contexts without requiring specialist infrastructure [3].

The combination of TB with other adjunctive modalities consistently outperformed any single tool used in isolation. When autofluorescence, CL, and TB were used together in a clinicopathological study, specificity improved beyond that of any individual tool [16]. The ViziLite Plus system, which combines chemiluminescent illumination with TB staining, has been reported to achieve sensitivity and specificity approaching 100% and 97.3%, respectively, in specific comparative evaluations [4]. TB also serves a practical surgical function: it is used intraoperatively to assess resection margins in OSCC cases, with evidence suggesting that it is less specific but more sensitive than frozen-section biopsy in detecting positive mucosal margins [11]. The concept of field cancerization, the presence of genetically altered tissue surrounding a primary tumor, further reinforces the rationale for TB deployment beyond the obvious primary lesion, as TB can reveal abnormal tissue in areas that appear clinically normal under white light [11].

Notwithstanding its strengths, TB functions most appropriately when clinicians interpret the results alongside the full clinical picture. A positive staining result in a lesion with local irritants should prompt removal of the causative factor and re-staining after two weeks before biopsy is mandated; however, a clinically suspicious lesion that stains negative should still be referred for biopsy, as false-negative results occur in heavily hyperkeratotic surfaces [2,8].

Limitations of vital staining and future directions

Despite its established utility, TB vital staining has well-characterized limitations that constrain its independent diagnostic reliability. The most clinically relevant of these is the occurrence of false-positive results, which arise primarily from inflammatory mucosal conditions, including traumatic ulcers, lichen planus, and hyperplastic candidiasis, all of which can bind the dye non-specifically due to disrupted epithelial integrity [2,12]. Conversely, false-negative results occur in lesions covered by a thick keratinized surface layer that impedes dye penetration into the underlying dysplastic epithelium [11]. These opposing error modes indicate that TB does not generate sufficiently sensitive or specific results across all clinical contexts to supplant histopathological examination.

Operator dependence is the second major constraint of this technique. The interpretation of staining intensity and distribution is qualitative, relying heavily on the examiner's experience to distinguish pathological retention from non-specific staining artifacts [1]. Although partially mitigated by standardized protocols, inter-observer variability in staining interpretation introduces uncertainty, particularly in general practice settings where TB exposure may be limited [9]. The mutagenicity of stained cells under high-energy irradiation has also been raised as a theoretical concern, given TB's reactivity with RNA; however, this has not been demonstrated clinically at the concentrations and frequencies used diagnostically [10].

These limitations have motivated the development of a new generation of complementary technologies. The "Oral-O-Scope" prototype, a non-contact reflection spectroscopy device, exploits the TB-DNA interaction at the molecular level to generate quantitative spectral deconvolution data that distinguish monomeric TB binding, characteristic of malignant cells, from aggregated non-specific binding found in normal and inflammatory tissues [9]. This approach has the potential to overcome the most problematic aspect of conventional TB staining, namely the dependence on colorimetric clinical judgment, by converting qualitative observations into objective numerical outputs. Artificial intelligence-assisted interpretation of TB-stained images represents another potential emerging direction, with the prospect of training machine-learning models on large datasets of confirmed staining patterns to reduce diagnostic subjectivity and support decision-making in resource-limited settings [3,4]. Multimodal integration, combining TB with autofluorescence, spectroscopy, or salivary biomarker analysis, offers the most promising near-term path toward overcoming the sensitivity-specificity trade-off that limits any single adjunctive modality [4]. Future research should prioritize large-scale, multicenter, prospective studies with standardized staining protocols, histopathological reference standards, and economic analyses to guide the evidence-based integration of TB into public and oral health screening frameworks [3].

## Conclusions

TB vital staining has a well-defined and evidence-supported role in the clinical evaluation of oral mucosal lesions. Its molecular selectivity for nucleic acid-rich dysplastic and malignant epithelium, combined with a simple, inexpensive, and chairside-accessible protocol, makes it a practically deployable adjunct across a broad range of dental settings, from specialist oral medicine clinics to primary care facilities in low-resource environments. Pooled evidence confirms that its diagnostic accuracy exceeds that of clinical visual examination alone and that its reciprocal sensitivity-specificity profile complements light-based modalities such as chemiluminescence and autofluorescence. Its highest clinical value is realized in three scenarios: identifying occult or underestimated high-risk lesions, guiding biopsy site selection in complex or multifocal presentations, and serving as a risk-stratification tool for general dental practitioners who encounter potentially malignant oral mucosal changes in routine practice. Limitations related to false positives from inflammatory conditions, false negatives in hyperkeratotic lesions, and operator-dependent interpretation mean that TB cannot substitute for histopathological diagnosis and should always be used as part of a structured diagnostic protocol rather than in isolation. Emerging technologies, including reflection spectroscopy, artificial intelligence-assisted staining interpretation, and multimodal diagnostic combinations, offer the prospect of overcoming these constraints and extending the clinical applicability of TB-based approaches. Dental practitioners who systematically incorporate TB vital staining, interpret results in the clinical context, and maintain a low threshold for targeted biopsy are best positioned to contribute meaningfully to the early detection imperative, which remains the most powerful intervention available for reducing oral cancer morbidity and mortality.
